# Reconciling the Neurophysiological and Cognitive Theories of Stimulus–Response Spatial Compatibility Effects: A Visual–Motor Dissociation Approach

**DOI:** 10.3390/vision9020034

**Published:** 2025-04-17

**Authors:** Elton H. Matsushima, Jose Antonio Aznar-Casanova

**Affiliations:** 1 Laboratório de Estudos do Comportamento Humano e Animal, Programa de Pós-Graduação em Neurociências e Neurologia, Faculdade de Medicina, Instituto de Psicologia, Universidade Federal Fluminense, Niterói 24210-201, Brazil; eh_matsushima@id.uff.br; 2 Faculty of Psychology, Department Section of Cognition, Institut of Neuroscience, Universitat de Barcelona, 08035 Barcelona, Spain

**Keywords:** visuomotor integration, visual pathways, S–R spatial compatibility, Stroop, frames of reference, visuospatial attention

## Abstract

This study investigated the differential impact of two visual dimensions (direction and spatial location) in two spatial Stroop tasks, where the relevant dimension for the response varied. Three studies compared the interactions between spatial compatibility and congruence effects on reaction time performances to infer how the dorsal pathway (DP) and ventral pathway (VP) of visual processing interfered with one another in processing relevant and irrelevant spatial information. This allowed us to bridge neurophysiological mechanisms with dual-process models of spatial compatibility. The participants responded from an avatar’s perspective, manipulated through rotations relative to the forward position, along with independent rotations of the avatar’s screen and keyboard. The results revealed two distinct response patterns: in the Direction Stroop, the performance was influenced equally by the relevant direction (VP) and the automatic processing of irrelevant location (DP); in the Location Stroop, the VP exerted minimal interference on the DP. Furthermore, the Only Keyboard rotation disrupted hand–eye coordination, modulating the DP interference on the VP in the Direction Stroop. These findings provide insights into the functional interaction of these visual pathways and their contributions to spatial compatibility effects, along with evidence for the dual-process model of spatial compatibility. Some issues about the separation of visual pathways are discussed based on our results.

## 1. Introduction

Stimulus–response (S–R) spatial compatibility experimental paradigms such as the Simon task [1] and the Spatial Stroop task [2] have been widely used to investigate action control, specifically, the ability to inhibit irrelevant information for a goal (see [3] for a review). S–R spatial compatibility effects are the differences in reaction times and errors under conditions where the relationship between stimulus and response is compatible (direct or natural) versus conditions where the relationship is incompatible (indirect or unnatural).

In a Simon task, observers are asked to respond with spatially arranged manual responses (e.g., the left and right keys on a keyboard) to the non-spatial features of a stimulus (e.g., the color of a target). Even though the spatial position of the stimulus is irrelevant to the task, observers respond faster and more accurately when the response location and the stimulus location are spatially matched (compatible mapping) than when they are not (incompatible mapping), the so-called Simon effect [1,2,4].

The Simon effect has been explained as a natural tendency to respond to the location of a stimulus [1]. Despite the stimulus location being irrelevant to the task, a spatial code is automatically activated to respond to its location, along with a spatial code to respond to the relevant dimension of the stimulus [3]. Response selection is slower when the two spatial codes differ (incompatible) compared to when they indicate the same spatial property (compatible).

In the case of Spatial Stroop task, observers are also asked to respond with spatially arranged manual responses in a task where the stimulus location is also irrelevant. The difference relies on the fact that the stimulus does convey spatial information (e.g., the words LEFT and RIGHT). The Spatial Stroop effect occurs when the response to a stimulus is faster and more accurate when the relevant feature of the stimulus is congruent with the response location and with the spatial location of the stimulus (e.g., the word LEFT appearing in the left visual hemifield and responded to by the left key) than when in an incongruent condition [3].

The spatial Stroop effect has been explained in a similar way [2]: both the relevant and the irrelevant stimuli generate response codes that are processed in parallel. When the spatial codes match (compatible), there is no conflict, but when they do not match (incompatible), the response code to the irrelevant stimulus must be inhibited before executing the response, increasing the response latency.

The most widely accepted models explaining S–R compatibility effects are dual-processing models [5,6,7], which distinguish between two response selection routes, one direct or automatic and another indirect or intentional. The automatic route depends on innate or overlearned associations, while the intentional route depends on recent S–R associations, defined by the task instructions. In Simon and Spatial Stroop tasks, the automatic route is activated by the stimulus location, while the intentional route is activated in a controlled manner to the relevant dimension of the stimulus. Therefore, observers should inhibit automatic responses when they do not match the intentional responses in non-corresponding conditions. The longer response times observed in the non-corresponding conditions in Simon and Spatial Stroop tasks are due to this response selection conflict [5,6,7].

A relevant observation is derived from the dual-processing model of the S–R spatial compatibility effects. In Simon and Spatial Stroop tasks, the automatic route involves the rapid and implicit processing of a stimulus’ spatial information, accomplished unconsciously without requiring attention, while the intentional route involves slower and explicit controlled processes that require overt attention, allowing the identification of the relevant property to respond according to the task instructions [8]. This parallel processing, an automatic processing of a stimulus’ spatial information alongside a controlled processing of other stimulus properties, is analogous to the dual-process model of visual perception [9,10,11].

The visual system is structured by two neurological pathways of visual processing, a ventral pathway (VP) connecting the striate cortex to the inferotemporal cortex (IT), and a dorsal pathway (DP), connecting the striate cortex to the posterior parietal cortex (PP). The latter is a rapid stream of visual processing of spatial properties for the visual control of actions, while the former is a slower stream of visual processing dedicated to object recognition and binocular vision [10,11,12]. The VP receives both parvo- and magnocellular inputs, thus processing detailed colored visual information and visual contrast and movement information, connecting in the IT cortex this visual representation to long-term memory to support object recognition. On the other hand, the DP receives magnocellular inputs, yielding faster processing of movement information in the PP, connecting to the motor cortices, to allow online visually guided behaviors, such as manual apprehension and walking [11].

Considering the parallelism between the dual-processing model of the S–R spatial compatibility effects and the dual-process model of visual perception, one may find evidence for a theoretical neurophysiological implementation of higher-level cognitive processing, investigating S–R compatibility tasks such as the Spatial Stroop task. The issue is whether the two parallel chains of processing (automatic and intentional) in S–R compatibility tasks can be neurophysiologically implemented separately in the two visual processing pathways, respectively, the automatic processing implemented in the DP and the intentional in the VP.

The present series of experiments sought to test this hypothesis. The initial study involved a comparative analysis of performance on two Spatial Stroop paradigms: the Direction Stroop and the Location Stroop. In the former, the relevant stimulus dimension was its shape, more specifically, the meaning of the stimulus. In this experimental condition, visual processing must be carried out in the VP in order to perform object recognition, thus engaging intentional processing or instruction-based response selection. In the Location Stroop task, the relevant dimension was the stimulus location or visual hemifield where the stimulus appears. This time, visual processing must recruit the DP to accomplish response selection, since location is the relevant dimension. The comparison between these tasks would provide an assessment of the influence on the performance of the irrelevant dimension, the stimulus location processed by the DP in the first experimental condition and the stimulus meaning by the VP in the second condition. The differential pattern of interferences would indicate if the spatial location was processed by a more rapid and implicit visual pathway (DP) and shape (meaning) or by a slower, more controlled visual pathway (VP).

The second experiment addressed a related issue. Humans have the ability to take the visual perspective of others [13,14] and also of an avatar [15,16]. When taking the visual perspective of an avatar in a virtual setting, some spatial dissociation might occur between the observer’s and avatar’s perspectives. First of all, because the egocentric perspective is the natural view, any other allocentric perspective imposes adaptation, increasing processing times [17,18].

If the avatar is rotated by 180°, just like in observing from a mirror, one must respond with a spatially correspondent key, which is contralateral to the avatar. Thus, the 180° rotation reversed the S–R relationships [19,20]. On the other hand, when looking in a mirror, observers have few difficulties in responding with the congruent behavior instead of with the anatomically congruent hand [21,22,23].

The correspondence effect from the observer’s perspective was reversed when the avatar was rotated by angles larger than 90°. Thus, ipsilateral responses for the avatar position were also faster than contralateral responses, demonstrating that observers correctly adopted the avatar’s perspective and, as expected, showed a S–R spatial compatibility effect under avatar rotations [16]. In the present experiment, observers responded to the Spatial Stroop task while taking the perspective of an avatar in a virtual experimental setting, which could be in a position rotated to the cardinal axis (east, south, and west) relative to the canonical facing forward position or egocentric perspective (the north perspective).

An additional manipulation, which was the rotation of the virtual devices, the screen and response keyboard, explored whether the S–R spatial compatibility effects would reflect the differences in processing between the VP and DP in the third study. In some trials, only the screen was rotated following the avatar rotation; in other trials, only the keyboard; and in others, both were rotated. According to our starting hypothesis, the virtual rotations of the devices should differentially modulate the interference effects of the irrelevant stimulus dimension. When the irrelevant dimension is the stimulus location (processed by the DP), virtual rotation should affect the interference on performance, while there should be no modulation when shape was the irrelevant dimension (processed by the VP).

## 2. Materials and General Methods

### 2.1. Participants

In total, 130 psychology students (80% women), aged 19 to 24 years old (mean, *M* = 20.9 years; standard deviation, *SD* = 2.3) from the Universidad Rovira i Virgili in Tarragona, Spain, took part in the experiment and were randomly assigned to six experimental groups responding to an online S–R spatial compatibility task. Two Spatial Stroop tasks (Direction and Location) and three rotations of virtual devices (Only Screen, Only Keyboard, and Both Devices) were the between-groups variables combined to determine the six experimental groups. Twelve participants were excluded for having less than 85% of correct answers and completing the task outside the time limits of less than 10 min and more than 20 min. The sample sizes were 60 participants (54 right-handed) for the Direction Stroop task (23 participants in the Screen-Only Rotation, 18 in Keyboard-Only, and 19 in Both Devices) and 58 (53 right-handed) for the Location Stroop (18 in the Screen-Only, 18 in Keyboard-Only, and 22 in Both Devices).

The participants were recruited at the university campus, where a preliminary description of the experiment goals were provided at invitation, along with how much time the experiment would take, that there were no risks, and that they would receive course credits for their participation, as well as the link to the internet application. After accepting, the participants accessed the application by the link, where they received the informed consent form with more detailed descriptions. The participants’ data were anonymized by an alphanumerical code.

### 2.2. Apparatus and Stimuli

Personal data, informed consent, instructions, training trials, and experimental trials were inputted to a Java script online application using the PsyToolkit program (version 3.6.2) [24,25]. The application is a lightweight program that can be reliably run in any modern internet browser and is executed only after being downloaded to participants’ computers, in order to avoid internet speed issues in RT measurement. To ensure the proper experimental settings of responses on a physical keyboard, the PsyToolkit program was prevented from being run on mobile devices (smartphones and tablets).

**Figure 1:**
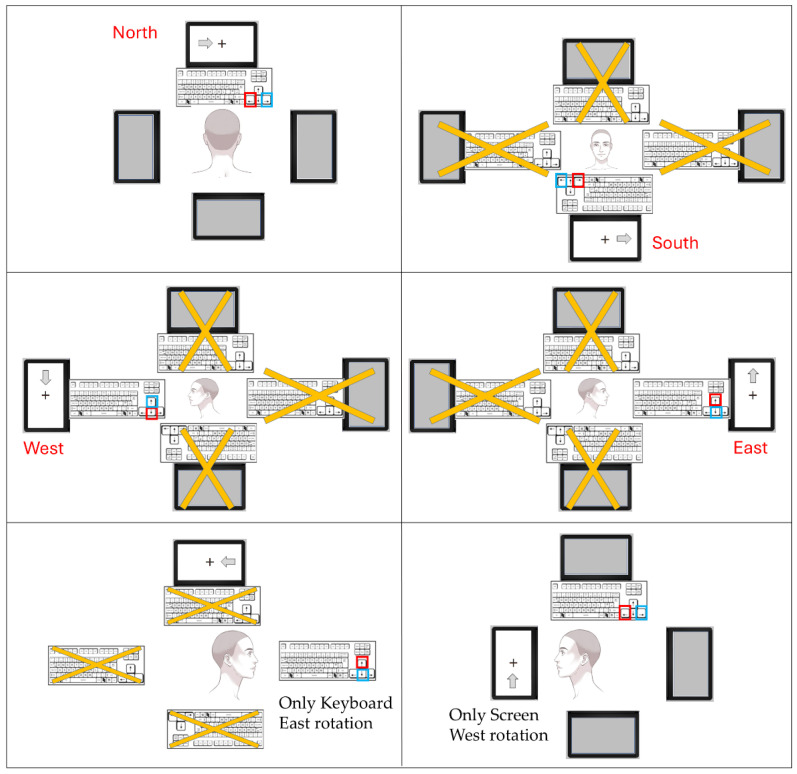
**Upper left panel**: screens presented in Study 1, depicting the north (canonical) rotation. **Upper right and middle panels**: screens presented in Study 2: south (mirrored, 180°), east, and west rotations (90° and 270°, respectively). **Lower panels**: two samples of screens presented in study 3, depicting an Only Keyboard east rotation and an Only Screen west rotation.

All the experimental screens were presented in full screen on a black background and the responses were pressing the standard keyboard arrows (the right, left, up, and down arrow keys) depending on the avatar rotation. The fixation target was a black cross in the screen center and the stimuli were arrows pointing to the left and to the right, whose onset was to the left or to the right of the fixation. Each experimental screen also presented an avatar, represented by a person’s head turned to observe a screen and a keyboard, according to the rotation to the north (the canonical position), to the south (the mirrored position, 180°), and to the east and west (counter- and clockwise 90° rotations). The screens also varied according to a device’s rotation: Only Screen, Only Keyboard, and Both Devices. In the Only Keyboard rotation, the observers had to consider only the rotation of the arrow keys, where the up and down keys take the place of left and right keys for the east and west rotations, and an inversion of keys in the south rotation (Figure 1). Additional figures can be found in the Supplementary Material Section.

### 2.3. Tasks

The participants responded to two Spatial Stroop tasks, a Direction Stroop task and a Location Stroop task, differing in the relevant stimulus dimension defining the selection of responses. They were asked to respond by pressing the left or right keys on the keyboard to the relevant stimulus dimension under compatible and incompatible instructions. The compatible instructions asked them to respond with a lateralized manual response with a key in the same direction, i.e., pressing the left key to a stimulus pointing to the left or a stimulus to the left of the fixation target (a black cross in the screen center). The incompatible instructions asked them to respond in a reversed mapping, i.e., pressing the right key to a stimulus pointing to the left or a stimulus to the left of the fixation.

The use of compatible and incompatible instructions allowed us to analyze the interaction between the congruence and compatibility: the congruence between the direction informed by the stimuli and the requested response and the compatibility between the stimulus location and the laterality of response. For a better understanding, see Figures in the Supplementary Material Section.

### 2.4. Direction Stroop Task

**Figure 2:**
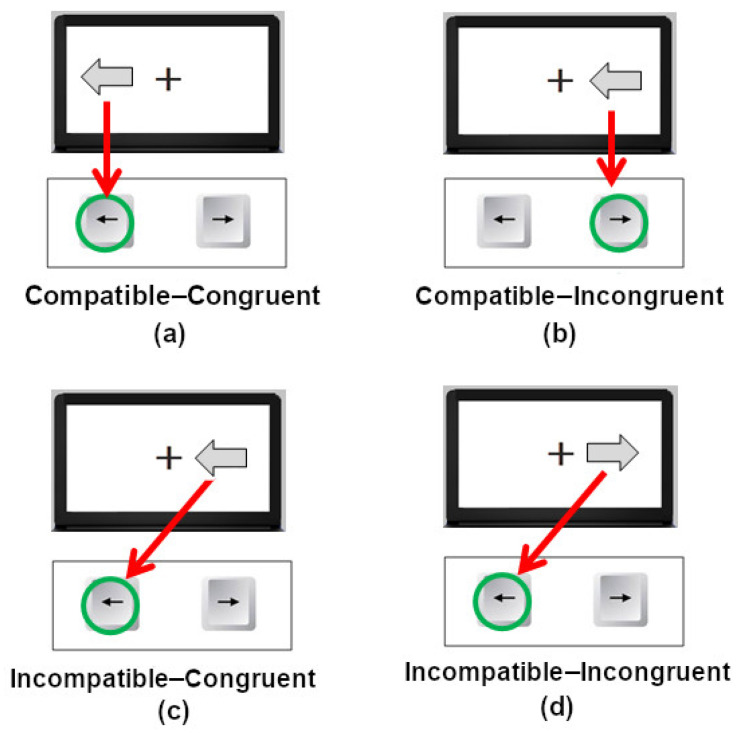
**Direction Stroop task**: The relevant stimulus dimension is the direction informed by the arrow, while the irrelevant dimension is its spatial position relative to the fixation. In the direct instructions, the observers must respond by pressing the left or right key to the same direction informed by the stimulus (left or right arrow), ignoring its location, either to the left or to the right of the fixation (Panels (**a**,**c**)). In the inverse instructions, the mapping of the stimulus dimension and response is reversed, e.g., pressing the right key to a left arrow and vice-versa (Panels (**b**,**d**)). **Location Stroop task:** The relevant stimulus dimension is the stimulus location, to the left or to the right of the fixation, while the arrow direction is irrelevant for the response selection. In the direct instructions, the observers must respond by pressing the left or right key to the stimulus location (left or right of the fixation), ignoring the arrow direction (Panels (**a**,**b**)). In the inverse instructions, the mapping of the stimulus dimension and response is reversed, e.g., pressing the right key to a stimulus to the left of the fixation and vice-versa (Panels (**c**,**d**)).

The participants had to consider the shape, more specifically, the direction of an arrow (left or right arrow), which would appear either to the left or to the right of the fixation target, and press either the left or right arrow keys on their keyboard, depending on the instructions (Figure 2). The spatial location of the stimulus relative to the fixation is irrelevant to this task. One may argue that, in this task, we are inducing the involvement of the VP, since the observer must recognize the symbol, a left or right arrow, to be able to select the appropriate response.

Although the spatial location of the arrow is irrelevant to this task, it may produce an interference effect on performance. Considering that the spatial location of the stimulus is quick and effortless, processed by the DP, it would need to be inhibited to respond correctly to the relevant dimension of the stimulus.

The use of direct and inverse instructions allowed us to compare the interaction of two important factors for our objectives, which emerge from the test conditions of the task. On the one hand, there was the congruence (congruent and incongruent) between the meaning of the symbol and the requested response; on the other hand, the spatial compatibility S–R (compatible and incompatible). Figure 2 depicts these factors. All the S–R combinations comprised both the Spatial Stroop task conditions that were presented to observers, under direct and inversed instructions.

### 2.5. Location Stroop Task

The observers had to consider the spatial position of the arrow, which could appear to the left or right of the fixation target, to select the response, pressing the left or right keys (Figure 2), accordingly to the instructions. The meaning, i.e., the direction of the arrow, was now irrelevant to this task. The DP processing should be more involved in this task, since observers must only respond to the stimulus location, irrespective of its direction. In this case, one may expect a faster RT with less interference than the Direction Stroop task, considering that VP processing is slower and more controlled than DP processing.

### 2.6. Procedures

Before starting each experiment, after informed consent, the participants entered some basic personal data (age and sex) and responded to a manual dominance questionnaire (Edinburgh Handedness Inventory—Short Form [26,27]), which took approximately 1 min. Then, the participants had to read the instructions according to the randomly assigned task. The instructions were presented in several screens and were supported by images for a complete understanding of the task. Each group was given a specific set of instructions based on the respective task (Direction or Location) and rotation (Only Screen, Only Keyboard, and Both Devices).

The direct instructions in the Direction Stroop task asked the participants to “Imagine that you are seated facing a computer just in front of your eyes. In each trial, a shape (arrows pointing left and right) will appear on one of the four screens shown in the image below, oriented in the directions North, South, East or West. The others will be turned off. You must respond as if you were facing the screen with the stimuli, assuming the perspective of the avatar in the center of the image. Thus, if the screen is oriented towards the East, West or South, you must imagine that you have turned your head to face the screen. Your task will consist of pressing the left arrow key when the shape that appears is an arrow pointing left or pressing the right arrow key when the shape that appears is an arrow pointing right. You must press the keys with the index fingers of both hands”. The inverse instructions only differed by saying “Your task will consist of pressing the right arrow key when the shape that appears is an arrow pointing left or pressing the left arrow key when the shape that appears is an arrow pointing to the right.“ Then, examples of the stimuli and correct responses were shown for the canonical position (north) and for the south rotation.

In the Location Stroop task, the direct instructions only differed by saying “Your task will consist of pressing the left arrow key when the shape appears to the left of the cross or pressing the right arrow key when the shape appears to the right. You must press the keys with the index fingers of both hands”. The inverse instructions of the Location Stroop task only differed by saying “Your task will consist of pressing the right arrow key when the shape appears to the left of the cross or pressing the left arrow key when the shape appears to the right”. Then, again, examples of the stimuli and correct responses were shown for the canonical position (north) and for the south rotation.

Each participant performed 16 training trials, not included in the analyses. After the training trials, the participants responded to two blocks of 112 trials, the first under direct instructions and after a short resting interval; the second, under inverse instructions; thus, each participant responded to 224 trials. These 112 trials resulted from combining the four avatar positions (north, south, east, and west), two directions (left and right), two locations (left and right of the fixation), and seven repetitions.

Every trial lasted approximately 2–3 s, including the stimulus presentation and response time. Only the last seven repetitions of a trial were analyzed. Each trial began with the presentation of a representation of the virtual devices (screen and keyboard) with the avatar in the respective rotation. If the participant selected the wrong answer or took more than 8 s to respond, an incorrect answer message appeared, and the next trial was presented. If a correct answer was selected, a correct message appeared, and the next trial was presented.

## 3. Study 1—Spatial Stroop Tasks in Canonical Perspective

Although the participants responded to all of the virtual avatar rotations, we will separate the analyses to facilitate comprehension. This study aims to compare the blocks of trials from the Direction and Location Stroop tasks presented in canonical perspective (north) with the pattern of S–R spatial compatibility effects. Comparing the interactions between the S–R spatial compatibility effects and congruence effects allowed us to determine by indirect measures (speed of processing and accuracy of the response) how strong the interference of the irrelevant stimulus dimension was on the relevant one. In other words, we determined how the DP processing interfered with the VP processing and vice versa. The north perspective also works as a baseline to Study 2 to determine the changes on the interference effects produced by the virtual rotation of the avatar perspective.

Our hypothesis states that, if the spatial location is processed by the automatic route, processed by the DP, and the shape is processed by the intentional route, processed by the VP, one would expect greater interference effects in the Direction Stroop task than in the Location Stroop task. The irrelevant stimulus dimension would have a greater impact on the relevant dimension when it is processed by an automatic and faster visual pathway.

The full sample of participants was included in this analysis, since the participants from each device rotated conditions responded to the north orientation. Thus, there were 118 participants, 60 from the Direction Stroop task (23 in the Screen-Only Rotation, 18 in the Keyboard-Only, and 19 in Both Devices) and 58 from the Location Stroop (18 in the Screen-Only, 18 in the Keyboard-Only, and 22 in Both Devices).

The means of the median RT of each participant were entered as the dependent variables into the repeated measures analysis of variance (ANOVA) in the experimental design as follows: for the Direction Stroop, the three within-subjects factors of two congruences (congruent and incongruent) × two S–R spatial compatibilities (compatible and incompatible) × two stimulus locations (left and right); for the location Stroop, the three within-subjects factors of two congruences (congruent and incongruent) × two S–R spatial compatibilities (compatible and incompatible) × two stimulus locations (left and right). Multiple comparisons in the post hoc analyses used the Bonferroni correction to prevent Type I errors. The errors were not analyzed, because the average number of correct answers reached 95% and they strongly correlated to the RTs. The analyses were run on SPSS 18.0.

### 3.1. Results

#### 3.1.1. Direction Stroop Task

Our data follow a normal distribution, as assessed by the Kolmogorov–Smirnov normality test, thus allowing us to use the ANOVA on the means of the median RT. The ANOVA produced reliable differences only for the main factor of congruence, *F*_(1, 59)_ = 4.636, *p* = 0.035, and *η_p_*^2^ = 0.073, where the congruent conditions (mean = 1327.471 ms; standard deviation = 55.077) were faster than the incongruent conditions (*M* = 1417.579 ms; *SD* = 54.239), with a medium effect size. This result indicates that the participants were faster (90,108 ms) when the stimulus direction was congruent with the laterality of the response, i.e., responding with the left key to a left arrow. However, there was no spatial compatibility effect, *F*_(1, 59)_ = 3.129, *p* = 0.082, and *η_p_*^2^ = 0.050, since the participants responded equally quickly under the compatible and incompatible conditions, where the response side matched or not the stimulus location.

**Figure 3:**
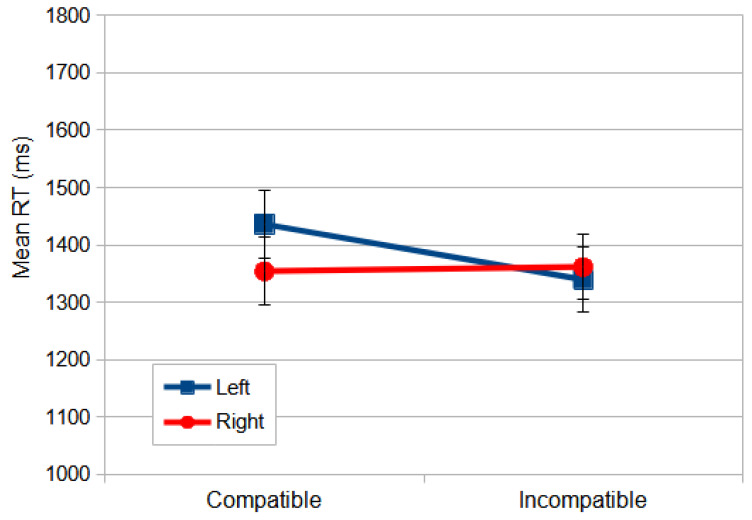
The mean RT (ms) for the Direction Stroop task in the north orientation as a function of compatibility and stimulus location. The error bars indicate standard errors of mean (SEMs).

Reliable differences were also found for the following interactions: congruence × compatibility, *F*_(1, 59)_ = 35.435, *p* = 0.000, and *η_p_*^2^ = 0.375, and compatibility × stimulus location, *F*_(1, 59)_ = 4.179, *p* = 0.045, and *η_p_*^2^ = 0.066. Considering the latter, the responses to a stimulus on the left in compatible conditions were slower than in all the other conditions (Figure 3). Responding with the non-dominant hand yielded slower responses, since most of the observers were right-handed (107 out of 118).

**Figure 4:**
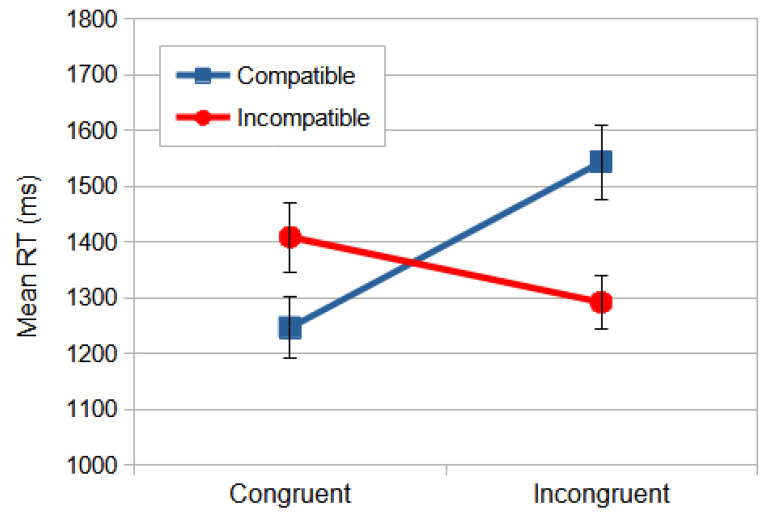
The mean RT (ms) of interaction congruence × compatibility from the Dimension Stroop task. Congruent conditions were those where the meaning of the stimulus was congruent to the laterality of the response. The compatibility was the matching between the stimulus position to fixation and the response key. The error bars indicate SEMs.

The significant effects of interaction congruence × compatibility are essential for the main goal of this study, since it allows us to measure the influence of the irrelevant dimension, stimulus location, on the relevant dimension, the meaning of the stimulus shape. One could argue that this influence is related to automatic and faster DP processing interference on slower and cognitively mediated VP processing. When the relevant dimension (direction) and the response are congruent, the observers were faster (164 ms) in the compatible condition than in the non-compatible condition (post hoc analyses, *p* < 0.001). However, when the relevant dimension and response were incongruent, the observers were faster (256 ms) in the non-compatible condition than in the compatible condition (*p* < 0.001) (Figure 4).

According to the dual-processing models, when the response codes of the relevant and irrelevant dimensions coincide, a facilitation for responses occurs, as in the case of the congruent–compatible and incongruent–incompatible conditions. However, interference would occur when the response codes did not match, as in the case of the congruent–incompatible and incongruent–compatible conditions. This conflict affected the response selection; thus, the observers must have inhibited the response code of the irrelevant dimension to be able to select the correct response [3].

#### 3.1.2. Location Stroop Task

A similar repeated measures ANOVA produced reliable differences for the main factors of compatibility, *F*_(1, 57)_ = 31.876, *p* < 0.000, and *η_p_*^2^ = 0.359, where the compatible conditions (*M* = 1421.047 ms; *SD* = 68.776) were faster than the incompatible conditions (*M* = 1220.116 ms; *SD* = 64.897) with a large effect size; congruence, *F*_(1, 57)_ = 11.101, *p* = 0.002, and *η_p_*^2^ = 0.163, where the congruent conditions (*M* = 1286.082 ms; *SD* = 65.214) were faster than the incongruent conditions (*M* = 1355.082 ms; *SD* = 65.346), also with a large effect size; and stimulus location, *F*_(1, 57)_ = 4.348, *p* = 0.042, and *η_p_*^2^ = 0.071, where the stimuli on the right side (*M* = 1294.274 ms; *SD* = 64.519) yielded faster responses than on the left (*M* = 1346.890 ms; *SD* = 66.814), with a medium effect size.

**Figure 5:**
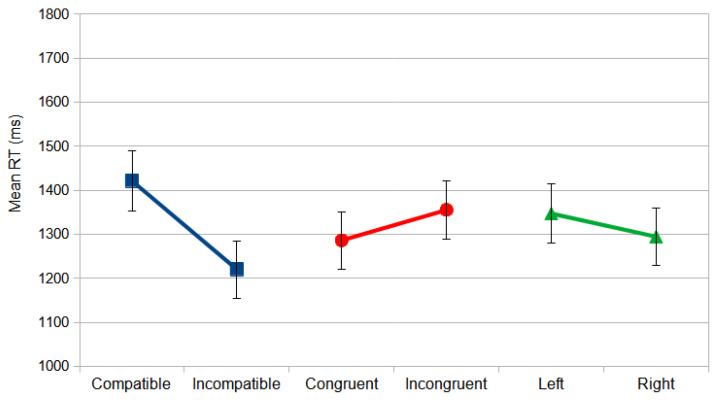
The mean RT (ms) for the Location Stroop task in the north orientation as a function of compatibility (blue), congruence (red), and stimulus location (green). The error bars indicate SEMs.

The significant effect of the compatibility analysis indicates that the observers responded faster (199 ms) when the relevant dimension (location) was incompatible with the response (Figure 5). This is called the Inverse Simon effect [3,28], when an irrelevant dimension of the stimuli interferes with the compatible responses. This inverted effect affects mostly the conditions under direct instructions, since they are always spatially compatible, suffering greater interference than under the reverse instructions, which are always spatially incompatible.

Considering the significant congruence effect, the congruent conditions yielded faster responses (69 ms) than the incongruent. The observers responded quicker with the left key to a left arrow and with the right key to a right arrow than with the inverse mapping (Figure 5).

For the reliable effect of stimulus location, the participants responded faster (29 ms) to the stimuli on the right side than to those on the left (Figure 5). These results may be attributed to the fact that most participants were right-handed (N = 107, out of 118) and therefore responded faster with their dominant hand than with their non-dominant one.

**Figure 6:**
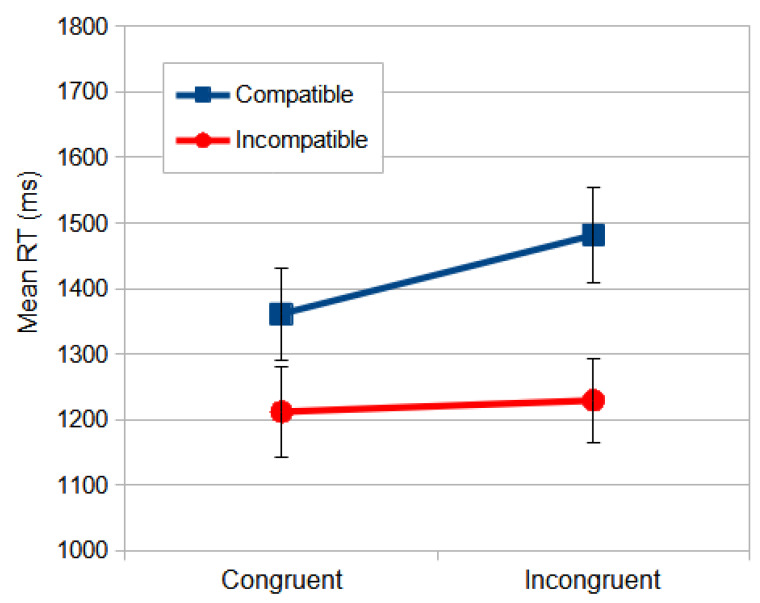
The mean RT (ms) of the interaction compatibility × congruence from the Location Stroop task in the north perspective. The congruent conditions are those where the meaning of the stimulus was congruent to the laterality of response. The compatibility was the matching between the stimulus position to the fixation and the response key. The error bars indicate SEMs.

However, the repeated measures ANOVA produced no reliable interactions, not even for the compatibility × congruence interaction, *F*_(1, 57)_ = 3.783, *p* = 0.057, and *η_p_*^2^ = 0.062. This allows us to suggest that, in the Location Stroop task, the interference of the irrelevant dimension (direction) on the relevant dimension (spatial location) was lower or absent. One may argue that the VP, which must be involved in processing the irrelevant dimension (direction), is slower than the DP, which was processing the spatial location, thus unable to interfere on much faster information processing (Figure 6).

According to the dual-process model, in compatible conditions, a facilitation effect was observed when the response codes of the relevant and irrelevant dimensions coincided, as in the compatible–congruent condition. Conversely, in compatible–incongruent conditions, where these response codes diverged, interference occurred. In incompatible conditions, however, the mismatch in response codes did not significantly affect reaction times (RTs). This suggests that the dissociation of the relevant and irrelevant dimensions is more readily achieved in incompatible conditions, likely due to the consistent contralateral mapping between stimuli and responses.

### 3.2. Discussion

This dataset served two purposes: (1) to establish the baseline performance for the Direction and Location Spatial Stroop tasks in a canonical, north-oriented perspective, where the avatar’s viewpoint aligned with the observer’s position; and (2) to provide evidence for the differential weighting of ventral (VP) and dorsal (DP) visual pathway processing in stimulus–response compatibility tasks by comparing the interference patterns from the relevant and irrelevant stimulus dimensions in the Direction and Location Stroop tasks.

In the Direction Stroop task, the irrelevant stimulus dimension is spatial location, processed by an automatic and rapid pathway, which we hypothesize to be the dorsal pathway (DP). Conversely, in the Location Stroop task, the irrelevant stimulus dimension is shape or arrow direction, processed by an intentional and slower pathway, requiring recognition, which we hypothesize to be the ventral pathway (VP). We propose that the interference from the slow, intentional process mediated by the VP will have a smaller impact on the rapid, automatic processing mediated by the DP, compared to the converse situation, where the DP exerts a stronger interference on VP processing.

The results support a differential interference pattern consistent with both the dual-process model [5,6,7] and our hypothesis regarding the ventral (VP) and dorsal (DP) visual pathways. In the Direction Stroop task, the irrelevant spatial location is processed rapidly by the DP, generating strong interference with the slow, controlled processing of the relevant arrow direction by the VP. Conversely, in the Location Stroop task, the irrelevant arrow direction, processed by the VP, did not significantly interfere with the relevant spatial location.

## 4. Study 2—Spatial Stroop Tasks in Different Avatar Perspectives

Within these experimental blocks, the participants’ visual perspective was manipulated by virtually rotating the avatar’s viewpoint, along with the associated virtual devices, screen and keyboard (see Figure 1). Building upon prior research demonstrating human capacity to adopt an avatar’s perspective when responding to stimuli [15,16], this analysis specifically examines the effects of rotating the avatar’s perspective to the south (180°), east (90°), and west (270°).

The aim of these virtual rotations was to investigate whether alterations in the frame of reference for the stimuli and responses would exert a greater influence on the dorsal pathway (DP), responsible for the spatial guidance of behavior, compared to the ventral pathway (VP), specialized in object identification and recognition. Consequently, we hypothesized that the interference pattern in the Direction Stroop task, where interference originates from the irrelevant spatial location processed by the DP, would be more significantly affected by rotations than the interference pattern in the Location Stroop task, where interference is induced by the irrelevant target meaning, processed by the VP.

### 4.1. Results

#### 4.1.1. Direction Stroop Task

First of all, the observers spent some time adopting another perspective, around 200 ms more than in the canonical perspective [28,29,30].

**Figure 7:**
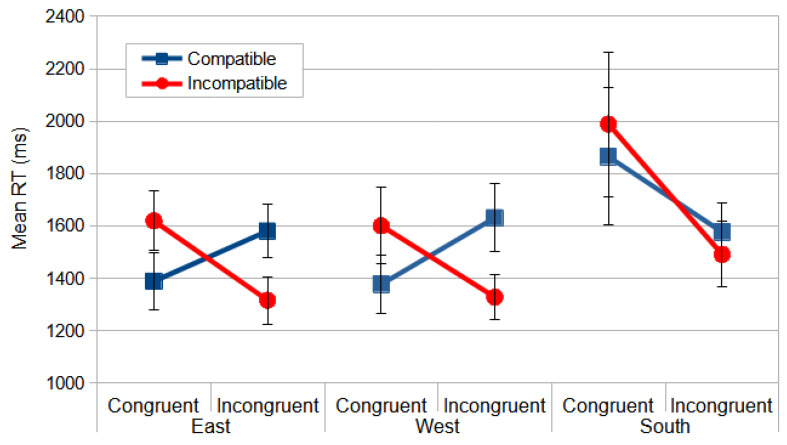
The mean RT (ms) of the interaction compatibility × congruence from the Direction Stroop task in east, west, and south rotations. Congruent conditions are those where the meaning of the stimulus was congruent to the laterality of response. The compatibility was the matching between the stimulus position to the fixation and the response key. The error bars indicate SEMs.

For the rotation of the virtual scene to the west, the repeated measures ANOVA for the individual means of the median RT following the experimental design of three within-subjects factors of two congruences (congruent and incongruent) × two compatibilities (compatible and incompatible) × two stimulus locations (left and right) did not reveal significant effects for the main factors of congruence, *F*_(1, 18)_ < 1, compatibility, *F*_(1, 18)_ = 1.688, *p* = 0.210, and *η_p_*^2^ = 0.086, and stimulus location, *F*_(1, 18)_ < 1. However, it showed significant effects for the interaction congruence × compatibility, *F*_(1, 18)_ = 20.754, *p* < 0.000, and *η_p_*^2^ = 0.536. The post hoc analyses revealed that, for the congruent conditions, the compatible trials were faster than the incompatible (*p* = 0.001), and, for the incongruent conditions, the incompatible trials were faster than the compatible (*p* < 0.000). In the compatible conditions, the congruent trials were faster than the incongruent (*p* = 0.001), but, in the incompatible conditions, the congruent trials were slower than the incongruent (*p* = 0.002). (Figure 7).

For the east rotation in the Direction Stroop task, the ANOVA for the same experimental design produced no reliable effects for the main factors of *congruence*, *F*_(1, 18)_ = 1.424, *p* = 0.248, and *η_p_*^2^ = 0.073, compatibility, *F*_(1, 18)_ < 1, and stimulus location, *F*_(1, 18)_ < 1, but indicated significant differences for the interaction congruence × compatibility, *F*_(1, 18)_ = 64.287, *p* < 0.000, and *η_p_*^2^ = 0.781. The same pattern of the post hoc analyses from the west rotation was found for the east rotation (Figure 7).

For the south rotation, the ANOVA produced no reliable effects for the main factors of compatibility, *F*_(1, 18)_ < 1, and stimulus location, *F*_(1, 18)_ < 1, but indicated significant differences for the main factor of congruence, *F*_(1, 18)_ = 4.989, *p* = 0.038, and *η_p_*^2^ = 0.217, and for the interaction congruence *×* compatibility, *F*_(1, 18)_ = 8.941, *p* = 0.008, and *η_p_*^2^ = 0.332. The incongruent conditions (*M* = 1533.605 ms; *SD* = 115.498) yielded faster responses than the congruent conditions (*M* = 1926.724 ms; *SD* = 268.335). The post hoc analyses revealed that only in the incompatible conditions were the incongruent trials faster than the congruent (*p* = 0.005).

Since there is a significant interaction with the significant main factor, we will concentrate our analysis on this interaction. In this mirrored perspective, compatible conditions imposed an inversion of the pattern from the canonical perspective, i.e., in the incongruent condition, the participants were faster than in the congruent one. This pattern in the south perspective also preserved the magnitude of the facilitation, around 300 ms. On the other hand, in the incompatible condition, the pattern found in the canonical perspective, incongruent faster than congruent, was magnified, increasing from 100 ms to 500 ms (Figure 7).

The east and west rotations maintained the effect of the interference of the irrelevant stimulus dimension on the relevant dimension as in the north perspective, although imposed an increase in RT due to a cognitive cost in processing time from the perspective taking. Since both rotations could be conceived as 90° rotations counter- and clockwise, the results agree with previous studies that showed an increase in RT with rotation [29,30,31], although they disagree with others that showed a delayed increase [16,32] only for angles greater than 90°. The same explanation based on the dual-processing model [5,6,7] suggested for the north perspective must apply to the east and west rotations. In the Direction Stroop, the irrelevant location would be processed quickly by the DP and generate a strong interference with the slow and controlled processing (VP) of the relevant dimension (the arrow direction).

The south rotation showed the same pattern of interference, where, in the congruent condition, the participants were faster for the spatially compatible condition, while, in the incongruent condition, they were faster in the incompatible condition. However, the significant effect of congruence shifted the pattern, increasing almost 300 ms in RT for the congruent conditions.

#### 4.1.2. Location Stroop Task

**Figure 8:**
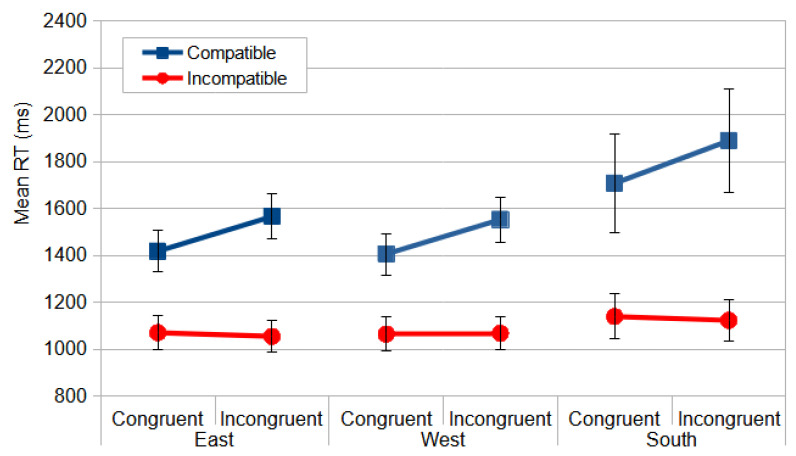
The mean RT (ms) of the interaction compatibility × congruence from the Location Stroop task in the west, east, and south rotations. Congruent conditions are those where the meaning of the stimulus was congruent to the laterality of response. The compatibility was the matching between the stimulus position to the fixation and the response key. rror bars indicate SEMs.

For the west rotation, the repeated measures ANOVA for the mean RT of three within-subjects factors of two congruences *×* two compatibilities *×* two stimulus locations revealed reliable effects for the main factors of compatibility, *F*_(1, 21)_ = 39.172, *p* < 0.000, and *η_p_*^2^ = 0.651, congruence, *F*_(1, 21)_ = 6.488, *p* = 0.019, and *η_p_*^2^ = 0.236, and the interaction compatibility *×* congruence, *F*_(1, 21)_ = 7.431, *p* = 0.013, and *η_p_*^2^ = 0.261. Since the interaction between both factors that presented significant effects was also significant, our analysis will focus on this interaction. In the compatible conditions, the observers were faster in the congruent conditions than incongruent, with no difference in the incompatible conditions (Figure 8).

For the east rotation, the ANOVA produced reliable effects for the main factors of compatibility, *F*_(1, 21)_ = 40.540, *p* < 0.000, and *η_p_*^2^ = 0.659, congruence, *F*_(1, 21)_ = 6.223, *p* = 0.021, and *η_p_*^2^ = 0.229, and the interaction compatibility *×* congruence, *F*_(1, 21)_ = 11.666, *p* = 0.003, and *η_p_*^2^ = 0.357. The same pattern of the interaction between the factors from the west rotation was found for the east rotation (Figure 8).

For the south rotation, the ANOVA revealed significant effects for the main factors of compatibility, *F*_(1, 21)_ = 21.181, *p* < 0.000, and *η_p_*^2^ = 0.502, congruence, *F*_(1, 21)_ = 10.108, *p* = 0.005, and *η_p_*^2^ = 0.325, and the interaction compatibility *×* congruence, *F*_(1, 21)_ = 14.755, *p* = 0.001, and *η_p_*^2^ = 0.413. Once again, the same pattern was found from the north, west, and east rotations. However, the magnitude differed from the other conditions. The south condition produced an increase of around 300 ms in RTs relative to the other rotations for the compatible conditions in the Location Stroop task (Figure 8).

### 4.2. Discussion

The observers’ responses in the block of trials under the west, east, and south rotations were separately analyzed. However, the results will be compared to the north or canonical perspective. This comparison should elucidate the processes related to perspective taking.

In summary, when the participants adopt the west or east perspectives in both the Direction and Location Stroop tasks, a response pattern similar to that observed in the north perspective is observed; thus, the same processes arose in the canonical perspective and in 90° rotations [16]. This suggests that rotating the devices by 90° induces the comparable effects of the DP and VP on each other as those obtained in the canonical perspective. The only difference is some deceleration of responses in the Direction Stroop task that can be conceived as the cognitive cost of adopting a new perspective [28,29,30] and a facilitation of incompatible responses in the Location Stroop task.

In contrast, this response pattern changed for the south perspective. This discrepancy is likely because, in this case, the relevant dimension, the semantic content, becomes irrelevant to the task. For the observers to make a decision in this perspective, they must apply a different response rule—one that is based on the principle that the opposite of the opposite side in the avatar vantage point is, in fact, the ipsilateral side, rather than the contralateral side as in the other perspectives. The evidence sustained that the observers would not struggle with S–R compatibility tasks in a mirrored perspective, i.e., they would have no difficulties responding with the congruent hand of the avatar instead of their own congruent hand [21,22,23]. However, another study found contradictory evidence, for the correspondence effect was reversed when the avatar was rotated by angles larger than 90° [16].

**Figure 9:**
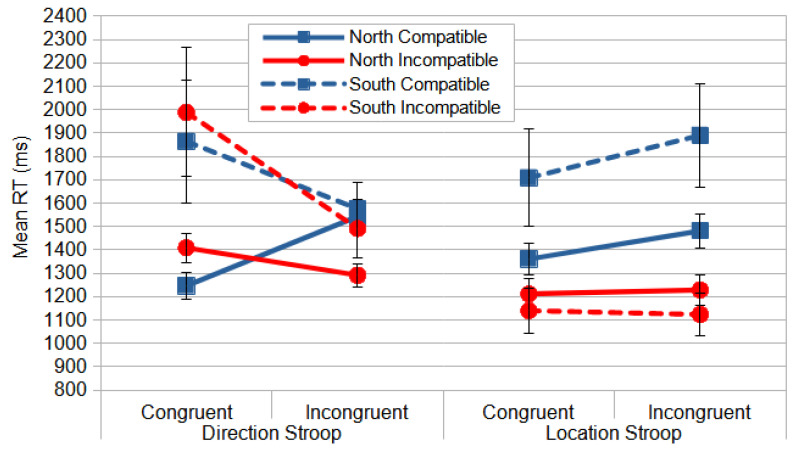
The mean RT (ms) of the interaction compatibility × congruence from the Direction Stroop task and Location Stroop tasks in the north and south rotations. Congruent conditions are those where the meaning of the stimulus was congruent to the laterality of response. The compatibility was the matching between the stimulus position to the fixation and the response key. The error bars indicate SEMs.

In our setting, an avatar at a 180° rotation would serve as a cue to inhibit the compatible responses, which would be activated by the automatic process. This inhibition seems to facilitate the alternative incompatible responses, which is the ipsilateral response from the avatar’s perspective, and delaying the compatible response, the contralateral response from the avatar’s perspective. This explains the south rotation response patterns in the Direction Stroop task, since this inhibition affects the responses in compatible conditions, inverting its trend, while keeping the same trend in incompatible conditions (Figure 9). In the Location Stroop task, the global pattern was preserved, but the compatible responses were largely inhibited, slowing them down by around 300–400 ms, and the incompatible responses were facilitated, in around 100 ms (Figure 9).

Focusing on the compatibility factor, collapsing all other factors, the compatible responses are slower than incompatible ones. This happens because, in the compatible condition, the spatial position is always on the same side as the manual response; thus, in order to respond to the relevant dimension, which would be, in half of the trials, the stimulus meaning, the irrelevant dimension must be inhibited. Since the automatic response to the irrelevant dimension is on the same response side of the relevant dimension, there would be interference and, consequently, a delay in latency. In the incompatible conditions, the relevant and irrelevant dimensions are easily dissociable, because they always appear on the other side to the manual response, resulting in less interference.

According to the dual-process model [5,6,7], in compatible conditions, when the response codes of the relevant and irrelevant dimensions align (the compatible–congruent condition), interference is reduced compared to conditions where they diverge (the compatible-incongruent condition), a pattern observed in the Direction Stroop task. However, in incompatible conditions, no significant differences were found between the congruent and incongruent conditions. It is plausible that the rapid processing of the relevant dimension, the stimulus location, by the dorsal pathway (DP) when presented contralaterally to the response (an incompatible condition) facilitated the response selection. This likely also prevented interference from the irrelevant dimension, the stimulus direction, which is processed by the slower ventral pathway (VP).

## 5. Study 3—Spatial Stroop Tasks in Different Device Configurations

Previous studies have provided some evidence for the distinct roles of ventral (VP) and dorsal (DP) pathway processing in Spatial Stroop tasks. However, the observed DP interference on VP processing within the Direction Stroop task did not definitively exclude alternative hypotheses. The primary objective of Study 3 was to achieve a clear dissociation between visual sensory processing (associated with the VP) and motor processing (associated with the DP). To accomplish this, two distinct device configuration conditions were incorporated into the experimental design. One experimental group responded to trials where only the screen was rotated across the four orientations (north, south, east, and west), while another experimental group responded to trials where only the keyboard was rotated across the same orientations (Figure 1, lower panels). These conditions were then compared to the data from the prior studies (Studies 1 and 2) where both the devices were rotated.

The independent manipulation of the screen and keyboard rotations was implemented to disrupt hand–eye coordination, thereby differentially affecting the ventral (VP) and dorsal (DP) pathway mapping and, consequently, reaction times (RTs) across the Stroop task types. Specifically, we sought to ascertain whether the discrepancies in RTs between the device rotation conditions—particularly between the trials with simultaneous screen and keyboard rotations and those with isolated keyboard rotation—would substantiate the hypothesis that the observed interference was predominantly driven by DP processing, given its established role in spatial orientation and visually guided motor actions.

The remaining experimental conditions were consistent with the prior research. The participants were presented with the four avatar orientations or perspectives (north, south, east, and west) in a randomized block design. Within each block, the trials were divided into two instruction sets. In the direct instruction set, the participants were required to respond with the left hand to a left-sided stimulus (defined by either direction or location) and vice versa. The other half were conducted under the inverse instructions, requiring participants to respond with the right hand to a left-sided stimulus (in terms of direction or location) and vice versa. These manipulations resulted in the within-subjects factors of congruence (stimulus–response spatial correspondence) and compatibility (stimulus–avatar perspective correspondence).

### 5.1. Results and Discussion

#### 5.1.1. Direction Stroop Task

The individual means of the median RT were submitted to a mixed factorial repeated measures ANOVA, with one between-groups factor of three device configurations (Only Screen, Only Keyboard, and Both Devices) and three within-subjects factors of four rotations (north, south, east, and west), two congruences (congruent and incongruent), and two compatibilities (compatible and incompatible).

**Figure 10:**
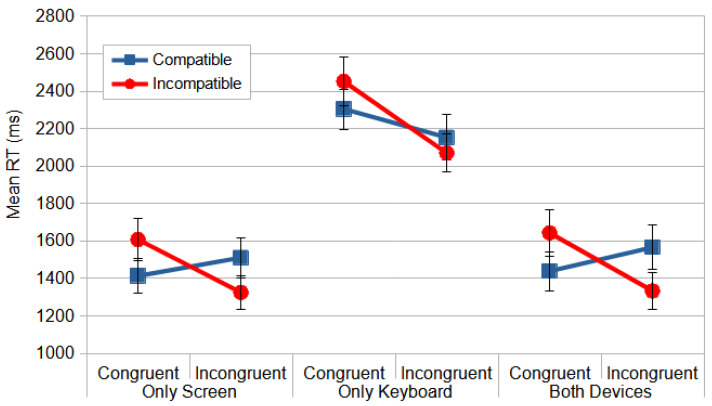
The mean RT (ms) for the Direction Stroop task as a function of device configuration, congruence, and compatibility. The error bars represent SEMs.

The overall results indicated that the Only Keyboard configuration had the greatest impact on the responses, with an increase in RT (Figure 10). This suggests that the keyboard displacement induced a greater dissociation between the visual module and the motor module. The ANOVA produced reliable differences for device configuration, *F*_(2, 57)_ = 17.998, *p* < 0.000, and *η_p_*^2^ = 0.387, and the post hoc contrasts, using the Bonferroni correction, only revealed a significant difference between the Only Keyboard and the other two device configurations.

The analyses also demonstrated significant effects for the main factors of *rotation*, *F*_(1.853, 105.625)_ = 38.481, *p* < 0.000, and *η_p_*^2^ = 0.403, and congruence, *F*_(1, 57)_ = 13.539, *p* = 0.001, and *η_p_*^2^ = 0.192, and for the interactions congruence *×* compatibility, *F*_(1, 57)_ = 76.313, *p* = 0.000, and *η_p_*^2^ = 0.572, rotation *×* congruence, *F*_(1, 57)_ = 51.540, *p* < 0.000, and *η_p_*^2^ = 0.475, and rotation *×* compatibility, *F*_(1, 57)_ = 9.166, *p* = 0.004, and *η_p_*^2^ = 0.139, in the Direction Stroop task.

Considering the interactions first, in the case of the interaction rotation *×* congruence, the RTs in the north perspective were faster in the congruent condition (1349 ms) than in the incongruent condition (1429 ms). However, in the other rotations, the responses were faster in the incongruent conditions than in the congruent conditions. Furthermore, in the case of rotation *×* compatibility, the RTs were faster in the north rotation, with even faster responses in the incompatible condition (1370 ms) than the compatible (1410 ms), compared to all the other conditions. RT was the slowest in the incompatible condition in the south rotation (1908 ms).

In the most relevant interaction, congruence *×* compatibility, the observers were faster for the congruent–compatible (1719 ms) than the congruent–incompatible (1901 ms) condition (*p* < 0.001) and were faster for the incongruent–incompatible (1576 ms) than the incongruent–compatible (1744 ms) condition (*p* < 0.001) (Figure 10). This indicates that, as predicted by the dual-process model and the two visual pathways model, when the response codes of the relevant and irrelevant dimensions align (the congruent–compatible or incongruent–incompatible condition), the dorsal pathway (DP) exerts less interference on the ventral pathway (VP) compared to conditions where they diverge (the congruent–incompatible and incongruent–compatible conditions).

The analysis of the significant main factors revealed that the north perspective yielded a significantly faster RT (1389 ms) (*p* < 0.0125); in contrast, the other perspectives, west (1859 ms), east (1827 ms), and south (1862 ms), did not differ significantly from one another (*p* > 0.05). This indicates that adopting a non-canonical perspective imposed a processing cost of nearly half a second, potentially due to additional cognitive processing and/or increased difficulty in response selection.

#### 5.1.2. Location Stroop Task

Consistent with the observations in the Direction Stroop task, the isolated keyboard rotation exhibited the most significant disruption of oculomotor coordination. A repeated measures ANOVA, conducted on the means of the median RT following the experimental design of the Direction Stroop task analyses, revealed a significant main effect for the between-subjects factor of device configuration, *F*_(2, 55)_ = 28.393, *p* < 0.000, and *η_p_*^2^ = 0.508. The post hoc comparisons, using the Bonferroni correction, revealed a significant difference only between the Only Keyboard and the other two device configurations.

**Figure 11:**
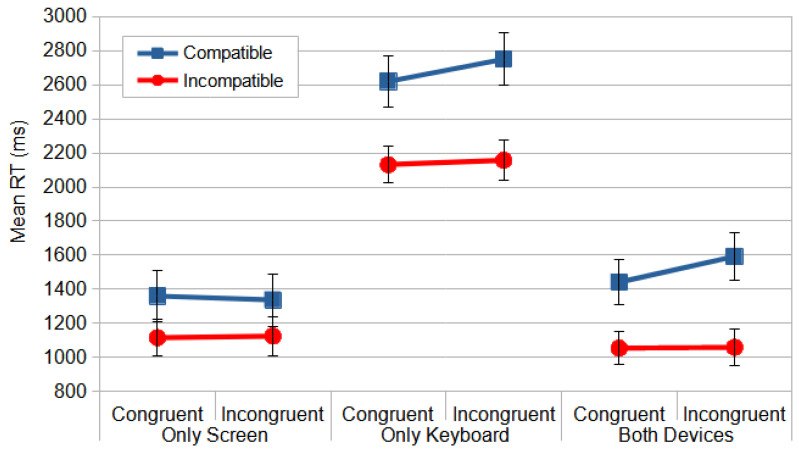
The mean RT (ms) for the Location Stroop task as a function of device configuration, compatibility, and congruence. The error bars represent SEMs.

Considering the within-subjects analyses, the most relevant interaction would be the three-way interaction between device configuration, compatibility, and congruence, *F*_(2, 55)_ = 4.464, *p* < 0.016, and *η_p_*^2^ = 0.140, since the four-way interaction was non-significant, *F*_(2, 55)_ = 1.031, *p* < 0.363, and *η_p_*^2^ = 0.036. Compatibility also produced significant effects, *F*_(1, 55)_ = 104.850, *p* < 0.000, and *η_p_*^2^ = 0.656, as well as in its interaction with device configuration, *F*_(2, 55)_ = 5.202, *p =* 0.009, and *η_p_*^2^ = 0.159. All the device configuration conditions exhibited the inverse Simon effect observed in Study 1, characterized by interference from an irrelevant stimulus dimension on compatible responses [3,28]. The significant interaction revealed that the incompatible conditions yielded faster reaction times (RTs) for the Only Screen and Both Devices conditions. Conversely, the compatible–incongruent condition in the Only Keyboard condition resulted in the slowest RTs. The Only Keyboard condition produced a shift in the inverse Simon effect pattern, increasing RTs by approximately 1000 ms (Figure 11).

Another within-subjects factor with reliable effects was *rotation*, *F*_(1, 55)_ = 55.603, *p* < 0.000, and *η_p_*^2^ = 0.503, indicating that the north rotation yielding faster responses (1334 ms) than the other rotations, west (1748 ms), east (1733 ms), and south (1762 ms), which did not differ from each other, as the post hoc analyses revealed. Thus, the process of adopting a new perspective once again induced a cognitive cost of about 400 ms.

Congruence produced significant effects independently, *F*_(1, 55)_ = 10.365, *p =* 0.002, and *η_p_*^2^ = 0.159, and in interaction with device configuration, *F*_(2, 55)_ = 3.234, *p =* 0.047, and *η_p_*^2^ = 0.105. However, these effects were modulated by compatibility, since they only occurred in compatible conditions, as can be observed in Figure 11.

#### 5.1.3. Interference Effect in Study 3

In both tasks, the interaction between the relevant and irrelevant stimulus dimensions reached statistical significance, exhibiting large effect sizes. Consistent with the findings in Studies 1 and 2, the magnitude of the dorsal pathway (DP) interference on ventral pathway (VP) processing, as evidenced in the Direction Stroop task, was substantially greater than the VP interference on DP processing observed in the Location Stroop task. This differential pattern of interference effects was consistently replicated across all the experimental conditions. Importantly, the manipulations of the device configuration and avatar perspective resulted in a significantly more pronounced DP interference on VP processing in the Direction Stroop task compared to the VP interference on DP processing in the Location Stroop task.

## 6. Conclusions

The aim of this series of studies was to investigate whether the dual-process models of S–R spatial compatibility effects could be linked to the distinct visual pathways dedicated to object recognition and visually guided actions, specifically the VP and DP visual pathways. Integrating cognitive and neurophysiological levels of explanation would provide a more comprehensive account of the phenomena, reinforcing the relevance of both research lines. An experimental paradigm employing a spatial Stroop task, with stimulus meaning and spatial location as alternating relevant and irrelevant dimensions for response selection, provided the necessary dissociation to assess the separate contributions of ventral (VP) and dorsal (DP) pathway processing in close association with the automatic–direct and intentional–indirect routes proposed by dual-process models. In the Direction Stroop and Location Stroop tasks, the relevant information for response selection was the stimulus direction (i.e., the meaning of the stimulus shape) and the stimulus location in the visual field (relative to a central fixation target), respectively. Given that identical stimuli were presented to the left or right of the fixation target in both tasks, the stimulus direction and location served as the irrelevant dimensions for the Location Stroop and Direction Stroop tasks, respectively.

**Figure 12:**
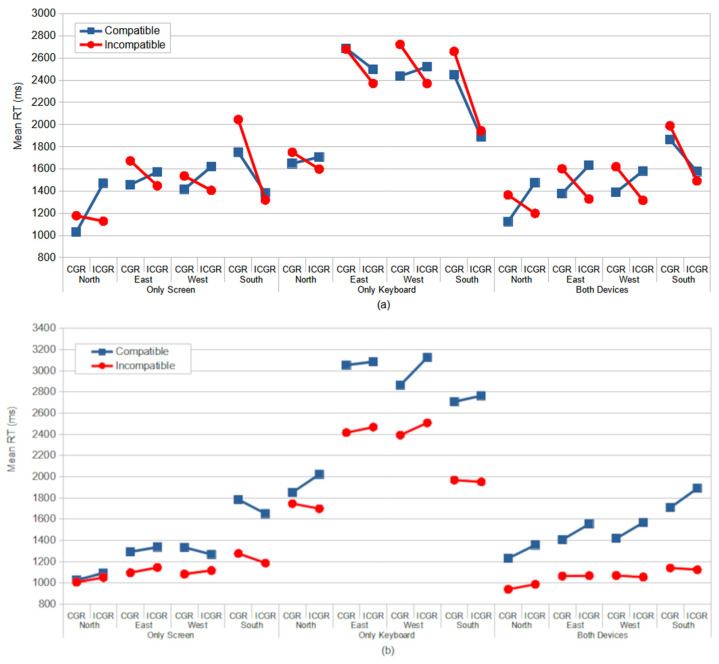
The mean RT (ms) for the Direction Stroop task (**a**) and Location Stroop task (**b**) as a function of device configuration, rotation, congruence (CGR—congruent; and ICGR—incongruent), and compatibility from Studies 1, 2, and 3.

Overall, our results revealed two distinct response patterns in the Direction Stroop and Location tasks. In the Direction Stroop task, when the congruent–compatible and incongruent–incompatible conditions yielded faster responses than the congruent–incompatible and incongruent–compatible conditions, except in the south rotations and in the Only Keyboard condition (Figure 12a). This general response pattern reflects the disruption of the controlled processes by automatic processes, as described by the dual-process model [5,6,7]. Conversely, in the Location Stroop task, the general response pattern was a facilitation of incompatible conditions over compatible conditions (Figure 12b) across all rotation and device rotation conditions. This inverted Simon effect [3,28] suggests that controlled processes exert minimal interference on automatic processes [2].

As previously described, the dual–process model of S–R spatial compatibility effects [5,6,7] posits an interactive relationship between automatic and controlled processes. Response selection is modulated by the congruence between response codes triggered by relevant and irrelevant stimuli. Automatic processing relies on innate or overlearned associations, whereas intentional processing relies on recently acquired S–R associations defined by task instructions. The participants are expected to inhibit automatic responses when they do not align with instruction–defined responses in non–corresponding conditions, resulting in longer RTs.

This dichotomy in the cognitive processing of S–R spatial compatibility effects parallels the established dichotomy in extrastriatal visual processing, wherein the DP is specialized for spatially oriented actions and the VP for object recognition [10,11]. Notably, DP processing aligns with the automatic route in dual–processing models, characterized by rapid, unconscious, and pre-attentive operations, while VP processing corresponds to the intentional route, marked by slower, conscious, and controlled mechanisms. The findings from this series of studies provide empirical support for this relationship between the dual–processing model of S–R spatial compatibility effects and the two visual pathways model of the visual system, thereby forging a robust connection between cognitive processing and neurophysiological accounts.

The differential effect of rotating the avatar’s perspective was expected to augment the influence of DP processing, which is crucial for spatial processing in support of motor control. This increase was hypothesized to be more pronounced in the Direction Stroop tasks compared to the Location Stroop tasks. Furthermore, rotations exceeding 90° were predicted to result in increased interference [16]. Overall, the rotations of the avatar’s perspective primarily induced a general slowing of performance, causing a shift in the response pattern without disrupting the inherent relationships between responses. Uniquely, an exception to this trend was observed in the south rotation.

The south rotation can be understood as a mirror perspective. While previous research has demonstrated minimal difficulty in adopting this perspective and responding accurately in spatial compatibility tasks [21,22,23], other studies have reported an inversion of the correspondence effect [16]. Our findings suggest that a mirrored perspective induces an inhibition of compatible responses, thereby facilitating alternative incompatible responses, specifically the ipsilateral response from the avatar’s perspective. This effect resulted in an inversion of the trend observed in the compatible conditions within the Direction Stroop tasks and either a slowing of the compatible conditions or a facilitation of the incompatible conditions within the Location Stroop tasks.

The observed effects of avatar rotation do not provide unequivocal evidence for a clear dissociation of interference between DP and VP processing. While the rotations had a greater impact on interference generated in the Direction Stroop task, suggesting a significant influence of the DP on the VP, they also affected the performance in the Location Stroop task, albeit to a lesser degree. This latter finding implies that VP processing does indeed exert an influence on the DP. While it might be counterintiuitive that a slower processing in VP could influence a faster processing in DP, this interaction could be possible if it occurs at a later stage of observer response, such as decision-making process, that is essential for accomplish the task and involved prefrontal cortex processes [33,34].

The significant effect of the Only Keyboard rotation on the responses provided clearer evidence for the asymmetrical influence of DP and VP processing in spatial compatibility tasks. The Only Keyboard rotation disrupted hand–eye coordination and interfered with the coordinated functioning between the VP and DP. However, while the pronounced impact on RTs in the Only Keyboard condition was observed, it did not eliminate the interference effect of the irrelevant dimension on the relevant dimension, which remained greater in the Direction Stroop task than in the Location Stroop task.

It could be suggested that, in the Direction Stroop task, performance was equally dependent on the processing of the relevant stimulus dimension, the direction inferred from shape recognition (a VP process), and on dorsal pathway (DP) processing, given that a lateralized manual response must be selected, which is subject to a rapid and automatic influence from the irrelevant stimulus location. Conversely, in the Location Stroop task, performance was hypothesized to be predominantly determined by the rapid and automatic processing of stimulus location. However, instances of faster responses were observed in compatible–congruent conditions compared to compatible–incongruent conditions.

An alternative account posits that the observed differences between the visual neural pathways stem from distinct frames of reference, with DP processing operating within an egocentric frame and VP processing within allocentric frames [35,36]. All perspectives presented in this study would be conceptualized as coded within an allocentric frame of reference, given that they are the avatar’s perspectives and not the observer’s. However, the congruence between the observer’s and avatar’s perspectives in the north rotation might facilitate processing within an egocentric frame. Regarding the coding of manual responses, the task instructions may promote coding in allocentric terms, despite the inherent automatic and direct processes being implicitly coded in egocentric terms. Nevertheless, our results do not support this hypothesis, as substantial differences in the RT pattern between the north rotation and other rotations were not observed.

Despite the widely accepted theory of the visual system being structured into independent processing pathways, instantiated by the DP and VP, accumulating evidence suggests that a fundamental characteristic of these systems is their interactivity and interdependence [11,33,34,37,38], indicating that the DP and VP function in an integrated manner. Research investigating these interactions has demonstrated, for example, that in object recognition tasks, the prior active control of exploratory views reduces the RT for object recognition compared to passive exploratory views [39]; a delay in pointing responses toward a display with motion illusion disrupts manual responses [40]; and in egocentric walking tasks, a 12 s delay or an imagined target (both increasing cognitive processing load) results in the undershooting of egocentric walked distance, mirroring perceptual performance in verbal judgments [41]. Taken together, these findings unequivocally suggest that DP and VP processing interact to produce these performance patterns.

Neurophysiological evidence from event-related potentials during avatar perspective taking suggests the involvement of the TP450 component (or P3), which reflects the activity in connections between the occipital, parietal, and temporal lobes, particularly the temporoparietal junction (TPJ) [42,43]. While the TPJ is commonly considered part of the DP, alongside the arcuate and superior longitudinal fasciculi and the posterior superior temporal lobe, and extends to the inferior frontal gyrus and premotor cortex [44], it plays a role in various perspective–taking tasks, ranging from spatial perception to the theory of mind [45]. The TPJ is also frequently associated with target detection mechanisms, alongside the medial temporal cortex and the lateral prefrontal cortex [46]. The late frontal slow wave (LFSW) has been linked to managing conflict between perspectives following behavioral responses, indicating that the LFSW represents the executive control mechanism of the frontal cortex [42]. Other evidence related to the frontal cortex also supports its role in the interaction found between automatic and controlled processes, since the prefrontal cortex would be responsible for controlling excitatory and inhibitory processes during both VP and DP activation [37,38]. Consequently, our experimental paradigm likely activates a complex neural circuitry interconnecting the DP and VP areas with frontal areas, thereby suggesting a more intricate interaction than unidirectional interference within each Stroop task. However, this neural circuitry should be verified in experimental settings that record brain activity in these visual pathways, the striate to the posterior parietal cortex and the inferotemporal cortex, and then to the prefrontal cortex, while responding to these spatial Stroop tasks.

Finally, we must acknowledge several limitations of this present study that may raise questions regarding the validity of our results. First, our sample predominantly comprised female students (80%), which could have introduced a bias. Given that sex differences have been observed in S–R spatial compatibility tasks, further research is warranted to investigate potential performance variations between male and female participants [47].

Second, the online nature of our experiments introduced potential biases due to the participants using their personal computer devices with varying screens and keyboards. However, screen size was categorized into three groups—large, medium, and small—based on diagonal screen measurements. The participants were permitted to use any keyboard equipped with four orthogonal directional keys. The preliminary analyses of the Study 1 data revealed no significant effects of screen size or keyboard type on reaction times (RTs). Consequently, the impact of this limitation is likely minimal.

Third, the online format of our experiments introduced additional potential biases, including variations in lighting and potential noise and observers responding at different times of the day, that could affect performance. This latter factor represents the combined influence of circadian rhythms and chronotype on cognitive processes [48,49]. While the impact of these factors is not universally consistent, with some studies demonstrating minimal effects on RT [50], it can be argued that the observed variability in our online experiments did not significantly exceed that typically found in controlled laboratory settings. Consequently, these potential sources of bias are unlikely to have substantially affected our findings.

Another untested variable is the response style. Post–experimental interviews with some observers, who were students from our university, revealed two response styles: those emphasizing accuracy and those emphasizing speed of response. Although these response styles were not controlled and may have influenced performances, the results are consistent across conditions, with a rather enclosed distribution around the mean, which suggests that this influence was minimal, specially considering that there are always many interindividual differences in RTs.

## Data Availability

The datasets analyzed during this current study for all the experiments are available at the OSF Data repository: https://osf.io/b59zm/?view_only=2a7cf3e5c9a24049ad06f72489513cac (accessed on 11 april 2025).

## Supplementary Materials

The following supporting information can be downloaded at: https://www.mdpi.com/article/10.3390/vision9020034/s1, Additional figures depicting each experimental conditions were provided to enhance method comprehension and beeswarm figures were provided to assess data distribution as suggested by a reviewer.

## Abbreviations

The following abbreviations are used in this manuscript:

S–R	Stimulus–response
VP	Ventral pathway
DP	Dorsal pathway
IT	Inferotemporal
PP	Posterior parietal
RT	Reaction time
M	Mean
SD	Standard deviation
ms	Millisecond
TPJ	Temporoparietal junction
LFSW	Late frontal slow wave
