# Supplementary material for: Reconciling the Neurophysiological and Cognitive Theories of Stimulus–Response Spatial Compatibility Effects: A Visual–Motor Dissociation Approach
Source: Vision (Basel). 2025 Apr 17;9(2):34. doi: 10.3390/vision9020034 (PMC12015925; doi:10.3390/vision9020034)
Supplement: Supplementary file 1 [file vision-09-00034-s001.zip › Supplementary_FIGs_Study_1&2_Beeswarm.zip!/FIGURES_Suplementary-Material_Beeswarm_chart_Study1&2.pdf]

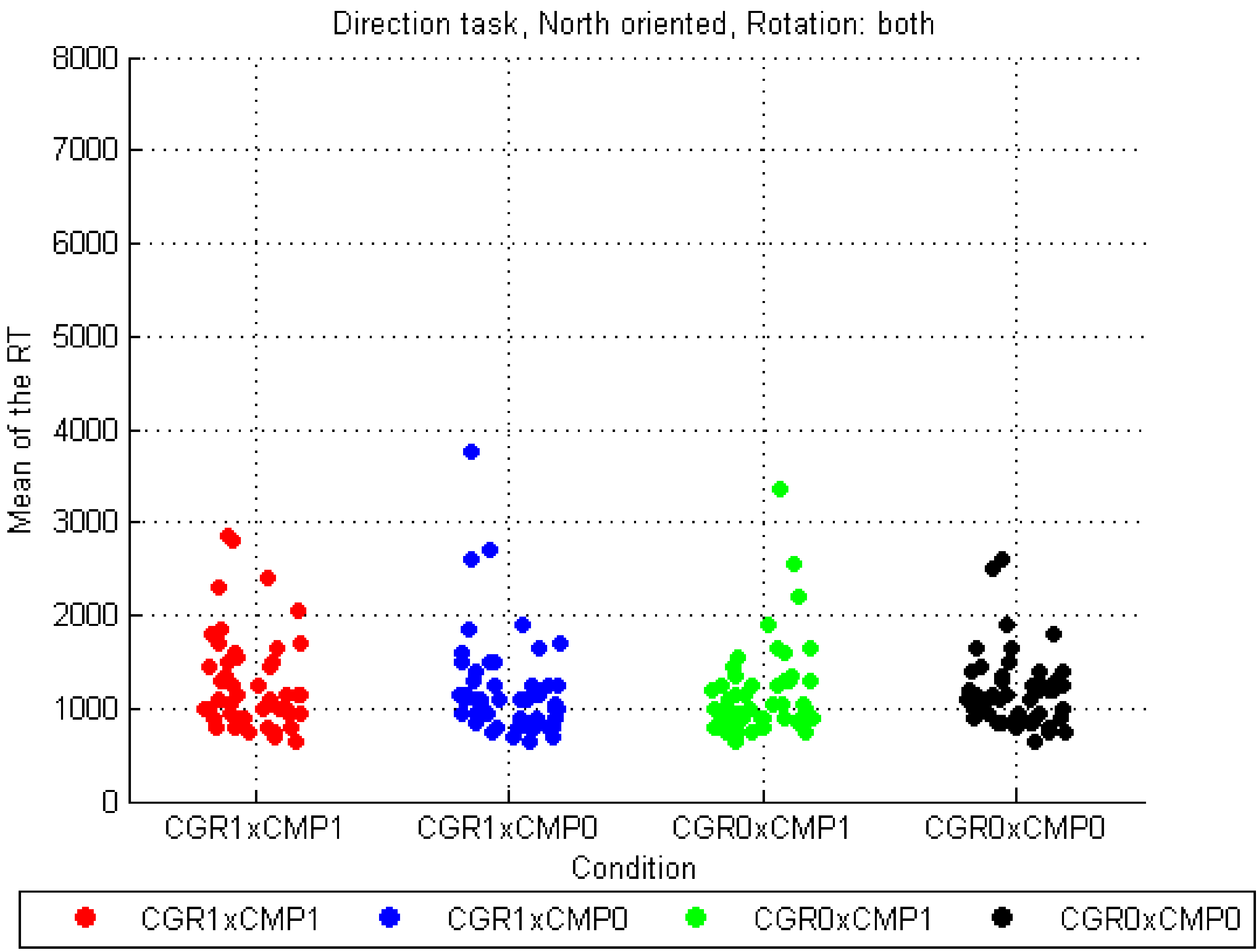

FIGURE 1 (Complementary-Material). Direction task; North oriented and both devices rotated

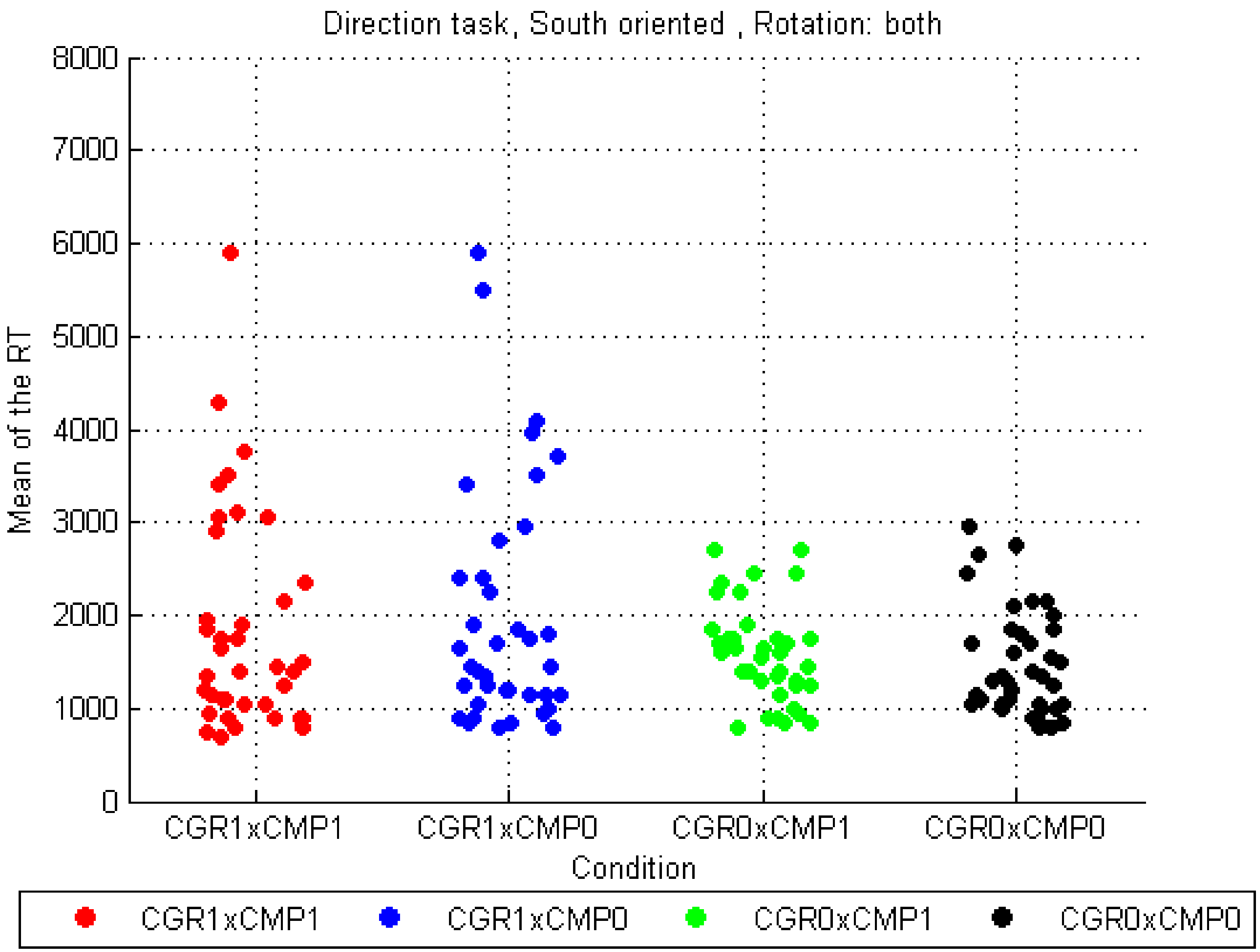

FIGURE 2 (Complementary-Material). Direction task; South oriented; and both devices rotated

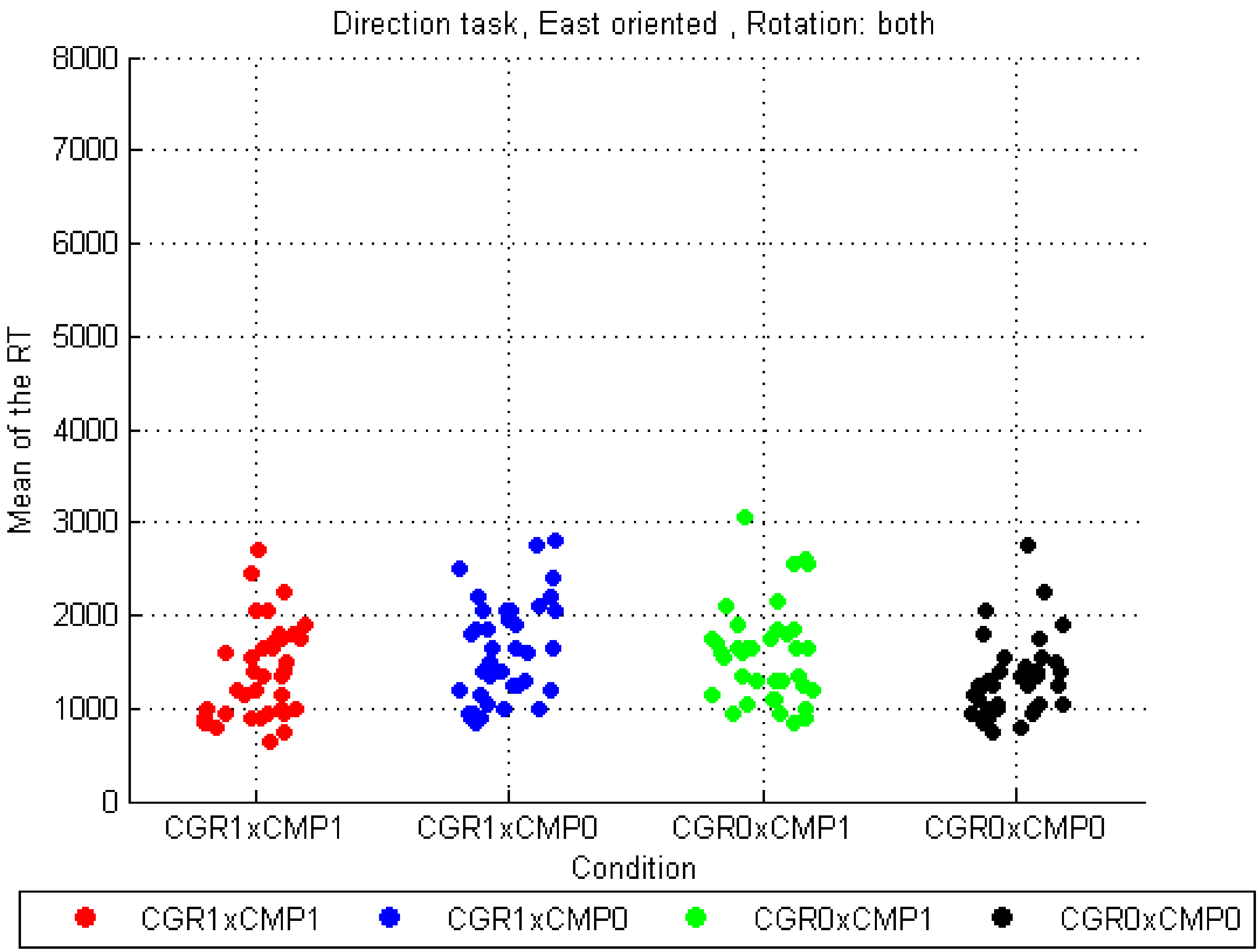

FIGURE 3 (Complementary-Material). Direction task; East perspective and both devices rotated

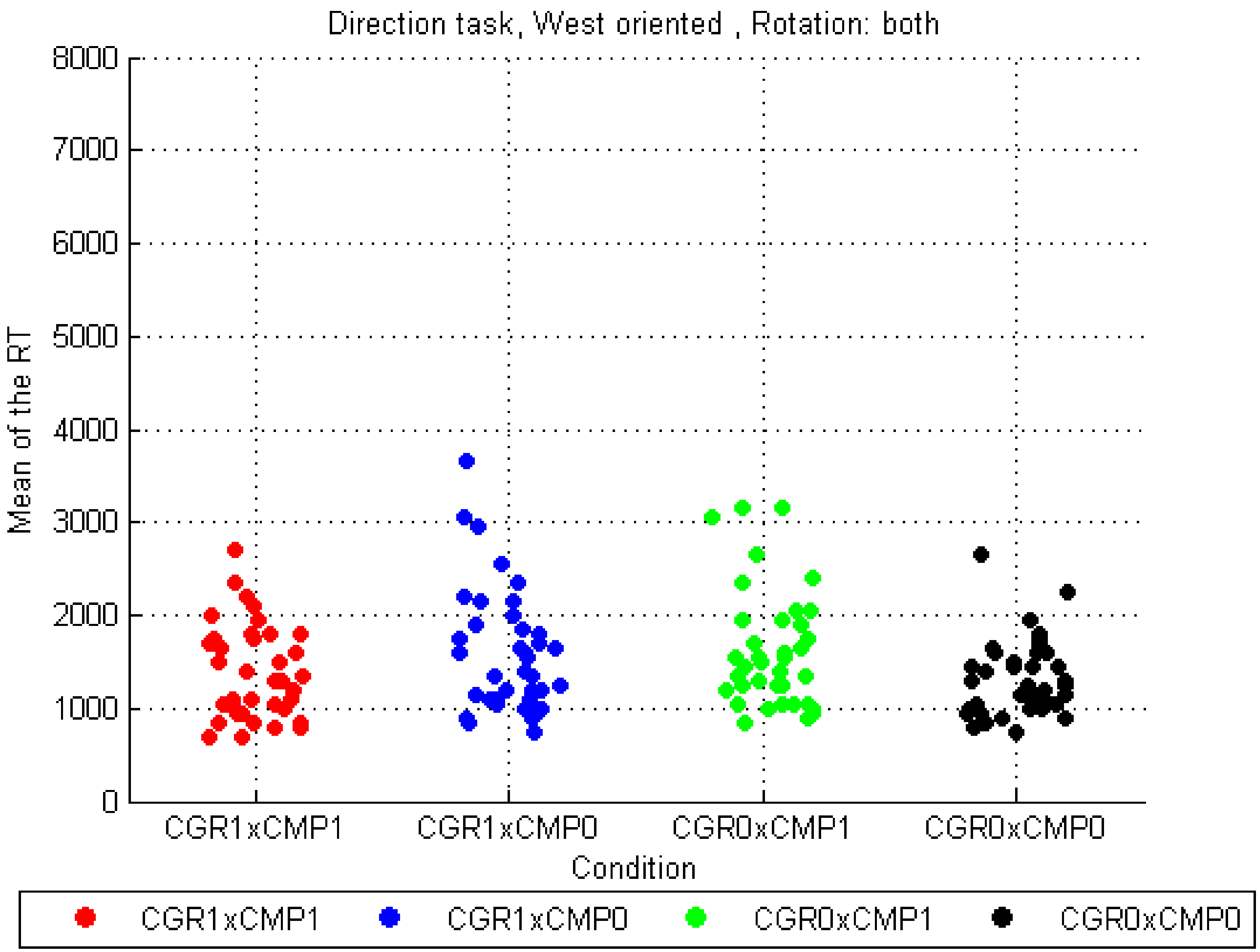

FIGURE 4 (Complementary-Material). Direction task; West perspective and both devices rotated

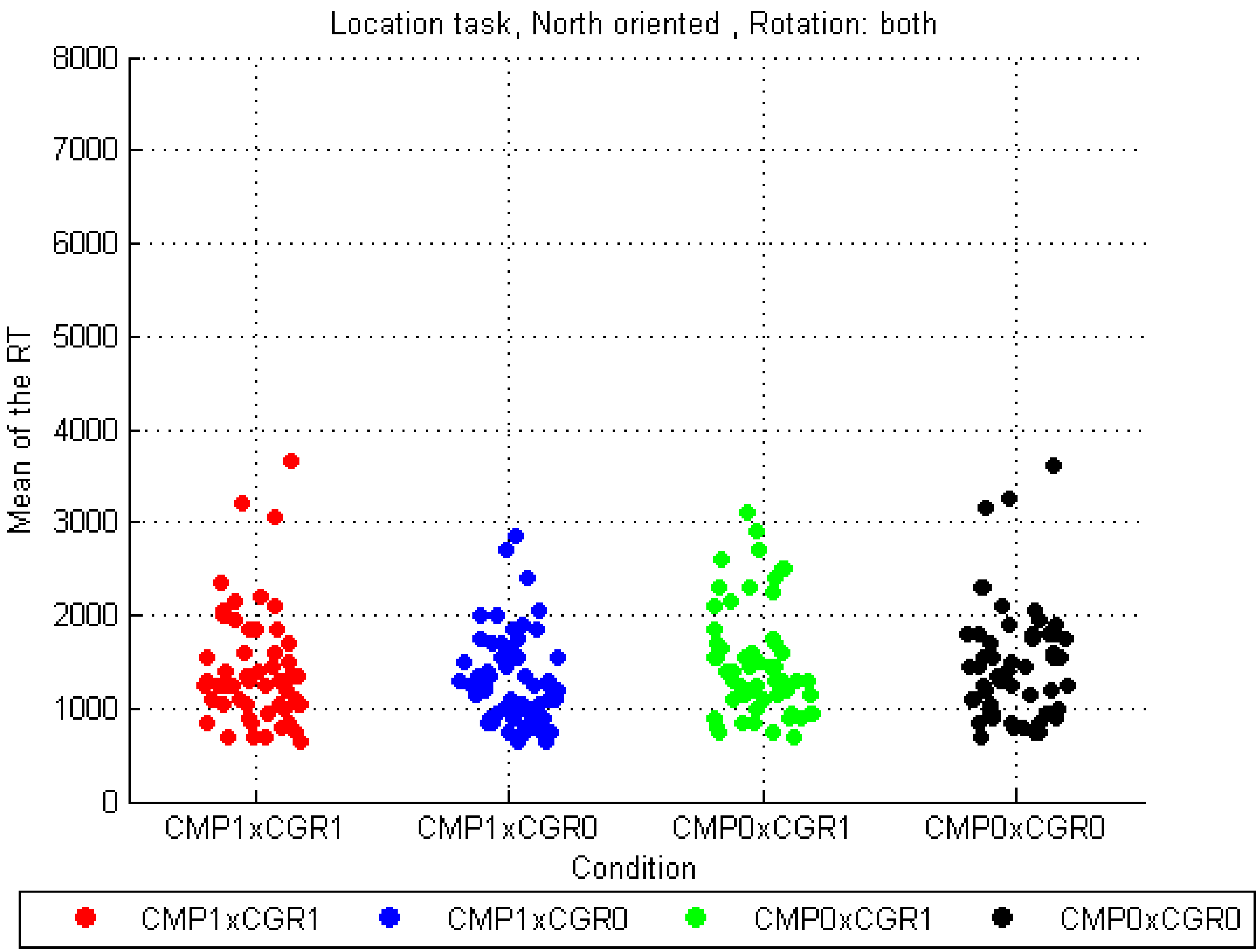

FIGURE 5 (Complementary-Material). Location task; North perspective and both devices rotated

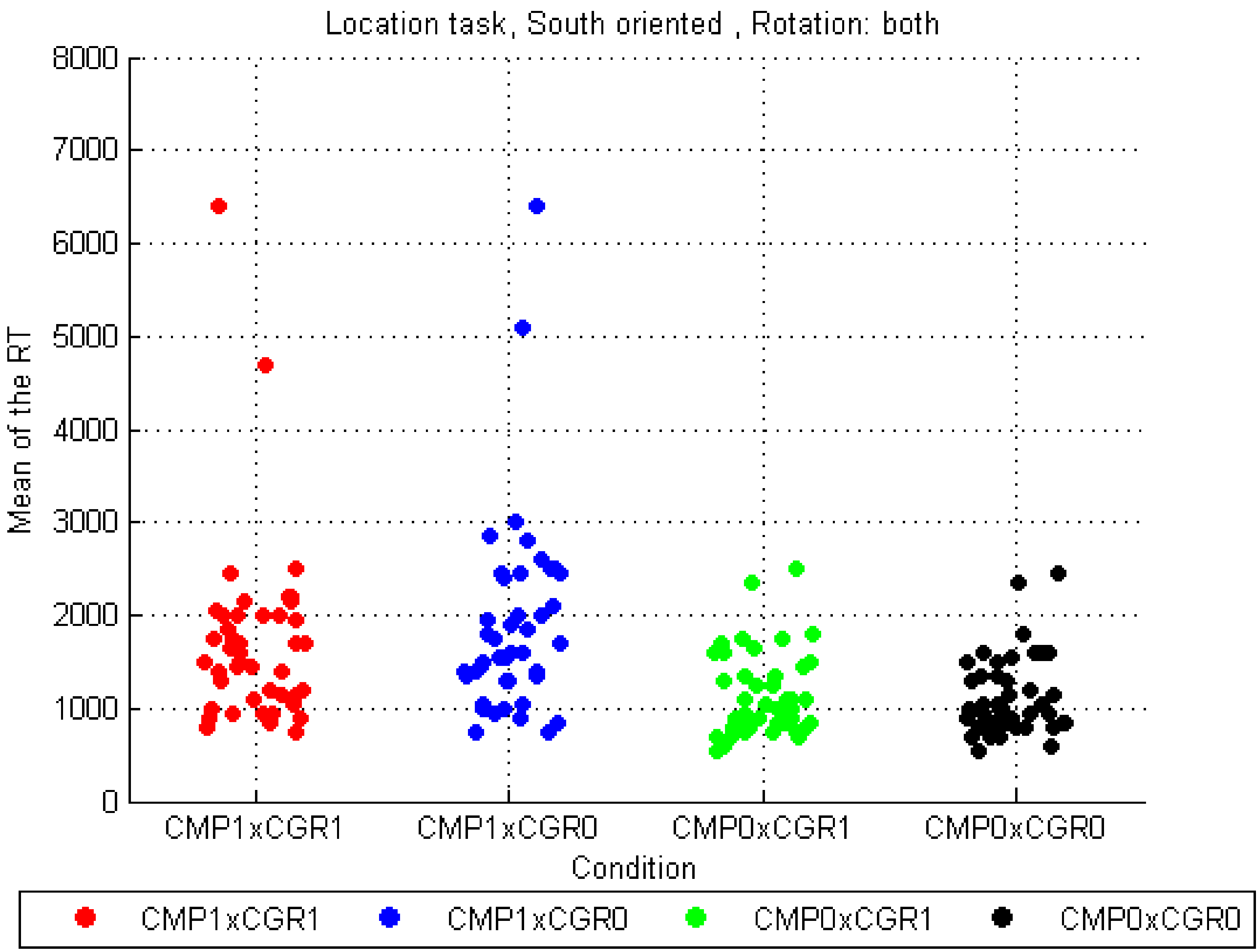

FIGURE 6 (Complementary-Material). Location task; South perspective and both devices rotated

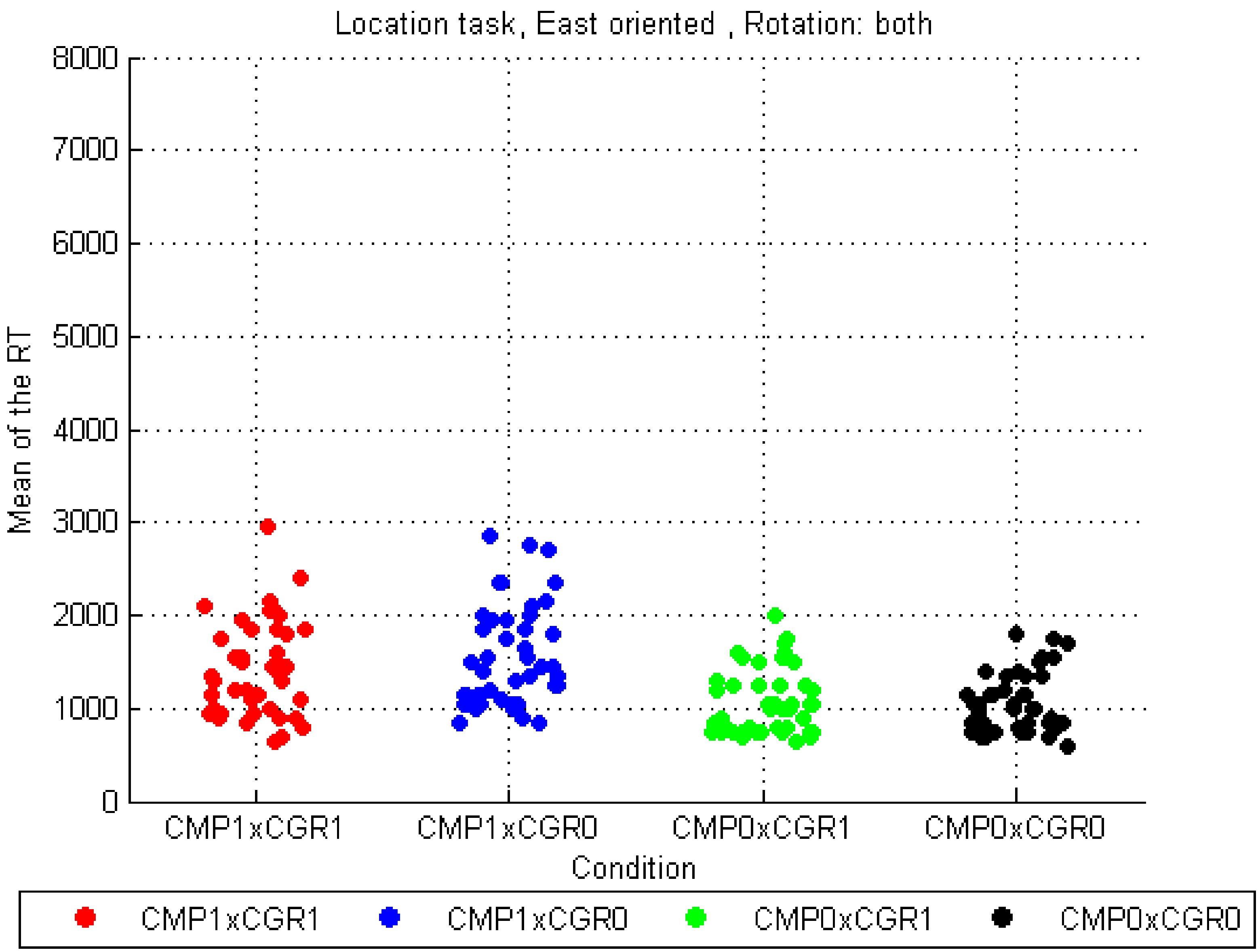

FIGURE 7 (Complementary-Material). Location task; East perspective and both devices rotated

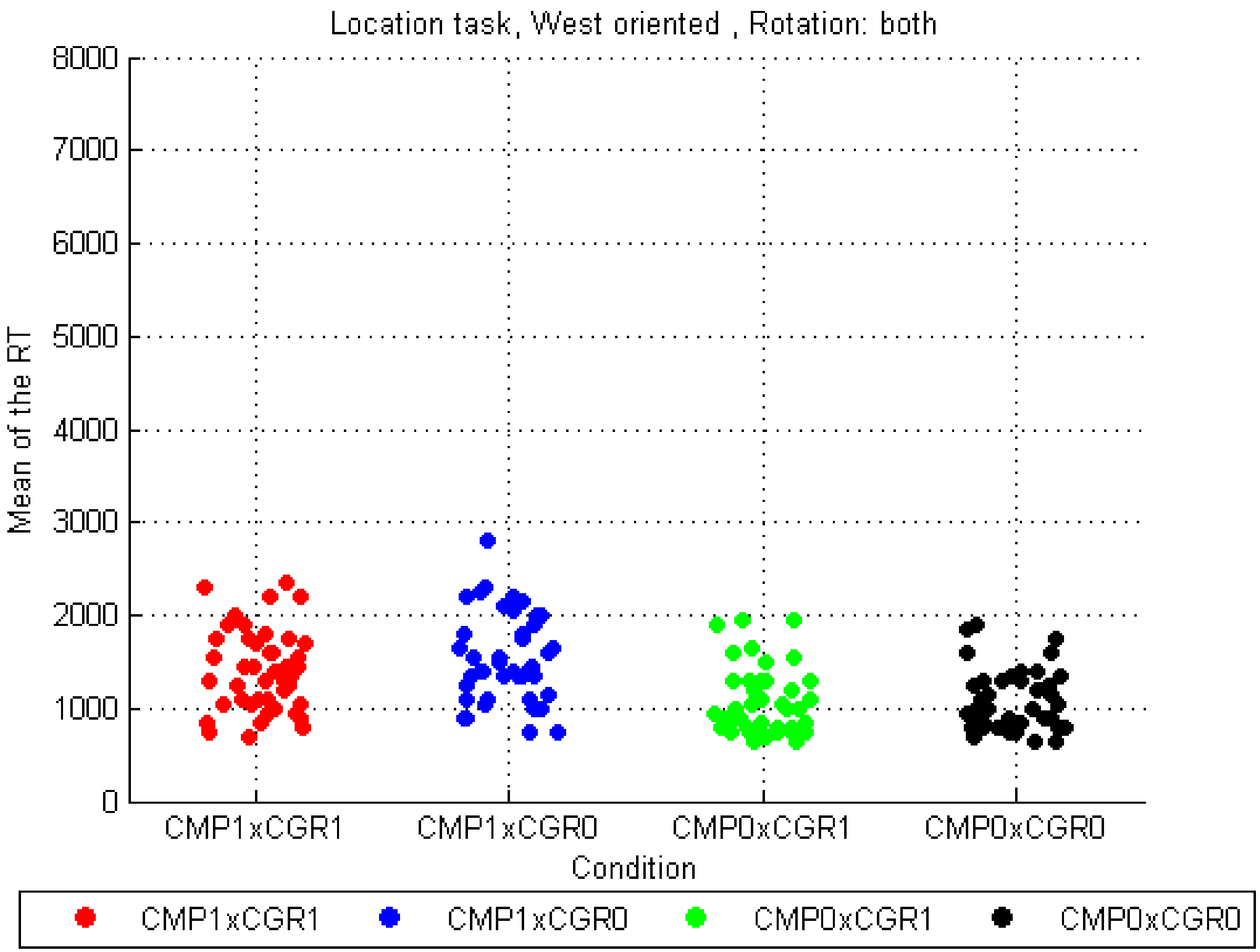

FIGURE 8 (Complementary-Material). Location task; West perspective and both devices rotated
